# TLR2/6 agonists and IFN-gamma treatment induces favorable immune cell recruiting signatures from melanoma associated with STAT1 and IL-32 signaling

**DOI:** 10.1186/2051-1426-2-S3-P225

**Published:** 2014-11-06

**Authors:** Ileana S Mauldin, Craig L Slingluff, Ena Wang

**Affiliations:** 1 Department of Surgery, University of Virginia, Charlotesville, VA, USA; 2 Transfusion Medicine, NIH, Bethesda, MD, USA

## 

Intralesional therapies offer promise to modulate immune signatures within melanoma and other cancers, either as monotherapy or as a component of combination immune therapy. We have shown that the TLR2/6 agonists (MALP-2 and FSL-1) and IFNγ induce human melanoma cells to synergistically produce T-cell attracting chemokine CXCL10. Despite the promise of inducing CXCL10 in the tumor microenvironment, IFNγ may induce negative immune regulatory processes, and TLR agonists have the potential to induce anti-apoptotic signaling in tumor cells. We hypothesized that synergy of IFNγ and TLR2/6 depends on STAT1 signaling, does not protect melanoma cells from apoptosis, and induces a more favorable immune signature than IFNγ alone.

To assess global effects of TLR2/6 agonist and IFNγ on melanoma, gene expression profiling of 4 melanoma cell lines was performed. They revealed that IFNγ treatment alone induced genes for CXCL9, and CXCL10 immune cell recruiting chemokines but also induced genes for negative immune regulators IDO and PD-L1 when compared to untreated cells. Comparison of TLR2/6+IFNγ stimulated melanoma cells to IFNγ stimulation alone showed induction of CXCL10, CXCL11, and C3 expression. Furthermore, genes encoding Treg-recruiting chemokines were not induced with TLR2/6 agonists+IFNγ; instead, profiles that promote Th1 and Th17 cells were observed. Gene profiling also demonstrated that IL-32 and STAT1 were induced by TLR2/6 agonists+IFNγ treatment when compared to TLR2/6 agonists or IFNγ treatment alone; we hypothesize that they may be the mechanistic mediators of the synergy between TLR2/6 agonists+IFNγ, since they have been shown to mediate this synergy for other TLR agonists.

Proliferation assays show that treatment with TLR2/6 agonists+IFNγ does not promote melanoma cell proliferation. Furthermore, viability assays demonstrate that TLR2/6 agonist+IFNγ treatment of melanoma does not hinder apoptosis. To address the specificity of TLR2/6 agonists we knocked down genes encoding for TLRs 1, 2 and 6 using siRNA. We find that TLR2/6 agonists may signal through TLR1, 2, and 6 suggesting that these putative TLR 2/6 agonists may also signal through TLR1/2 to mediate CXCL10 production in a wider range of melanoma tumors.

Collectively, our data suggest that TLR2/6 agonists induce favorable gene signatures which may promote immune cell infiltration of melanoma. Gene array analysis reveals that IL-32 and STAT1 may mediate the synergistic CXCL10 production observed from TLR2/6 agonist+IFNγ stimulated melanoma cells.

